# Drivers of hibernation in the brown bear

**DOI:** 10.1186/s12983-016-0140-6

**Published:** 2016-02-11

**Authors:** A. L. Evans, N. J. Singh, A. Friebe, J. M. Arnemo, T. G. Laske, O. Fröbert, J. E. Swenson, S. Blanc

**Affiliations:** Department of Forestry and Wildlife Management, Hedmark University of Applied Sciences, Campus Evenstad, NO-2418 Elverum, Norway; Department of Wildlife, Fish and Environmental Studies, Swedish University of Agricultural Sciences, SE- 90183 Umeå, Sweden; Department of Ecology and Natural Resources Management, Norwegian University of Life Sciences, Post Box 5003, NO-1432 Ås, Norway; University of Minnesota, Minneapolis, MN 55455 USA; Medtronic Inc, Mounds View, MN 55112 USA; Faculty of Health, Department of Cardiology, Örebro University, SE 70182 Örebro, Sweden; Norwegian Institute for Nature Research, Post box 5685 Sluppen, NO-7485 Trondheim, Norway; Université de Strasbourg, IPHC, Strasbourg, France; CNRS, UMR7178, Strasbourg, France

**Keywords:** Body temperature, Denning ecology, Metabolic inhibition, Physiological ecology, Thermoregulation

## Abstract

**Background:**

Hibernation has been a key area of research for several decades, essentially in small mammals in the laboratory, yet we know very little about what triggers or ends it in the wild. Do climatic factors, an internal biological clock, or physiological processes dominate? Using state-of-the-art tracking and monitoring technology on fourteen free-ranging brown bears over three winters, we recorded movement, heart rate (HR), heart rate variability (HRV), body temperature (T_b_), physical activity, ambient temperature (T_A_), and snow depth to identify the drivers of the start and end of hibernation. We used behavioral change point analyses to estimate the start and end of hibernation and convergent cross mapping to identify the causal interactions between the ecological and physiological variables over time.

**Results:**

To our knowledge, we have built the first chronology of both ecological and physiological events from before the start to the end of hibernation in the field. Activity, HR, and T_b_ started to drop slowly several weeks before den entry. Bears entered the den when snow arrived and when ambient temperature reached 0 °C. HRV, taken as a proxy of sympathetic nervous system activity, dropped dramatically once the bear entered the den. This indirectly suggests that denning is tightly coupled to metabolic suppression. During arousal, the unexpected early rise in T_b_ (two months before den exit) was driven by T_A_, but was independent of HRV. The difference between T_b_ and T_A_ decreased gradually suggesting that bears were not thermoconforming. HRV increased only three weeks before exit, indicating that late activation of the sympathetic nervous system likely finalized restoration of euthermic metabolism. Interestingly, it was not until T_A_ reached the presumed lower critical temperature, likely indicating that the bears were seeking thermoneutrality, that they exited the den.

**Conclusions:**

We conclude that brown bear hibernation was initiated primarily by environmental cues, but terminated by physiological cues.

**Electronic supplementary material:**

The online version of this article (doi:10.1186/s12983-016-0140-6) contains supplementary material, which is available to authorized users.

## Background

Hibernating mammals are good models for investigating the relationship between physiology, behavior, and environment, as hibernation patterns are important determinants of survival [1]. Studies of small hibernators suggest that climate variability might affect hibernation patterns and survival, as seen in the yellow-bellied marmot (*Marmota flaviventris*) exiting the burrows much earlier due to warming spring temperatures in spite of a consistent duration of snow cover [2]. Moreover, whereas energetics has been used as a predictor of a climate-change-associated northward expansion for certain species, including the little brown bat (*Myotis lucifugus*) [3], increased climate variability has been shown to decrease fitness in Columbian ground squirrels (*Urocitellus columbianus*), due to decoupling of environmental cues and food availability [4]. Mismatches between thermal and photoperiod cues pose a major challenge for hibernators [5]. Therefore, the phenology, interdependency, and chronology of physiological, behavioral, and ecological events, bracketing the hibernation period and their causative relationships, could provide important insights into individual plasticity to environmental challenges and predict whether climactic changes will cause mismatches between behavior, physiology, and food availability.

Many studies have focused on how environmental changes alter the phenology, morphological traits, and population dynamics of various species [4, 6], but have failed to account for the fact that phenotypic plasticity can modify model predictions [5, 7]. Indeed, because individual-centered physiological responses govern the link between environmental change and individual performance, scientists now recognize the necessity of incorporating physiological, behavioral, and demographic data into combined models [8]. However, this approach remains a challenge, due to the paucity of long-term physiological data collected on free-ranging animals, particularly in conjunction with behavioral and environmental data.

Much of the existing knowledge on the interplay between environment, physiology, and behavior in timing of denning comes from studies on small hibernating mammals; studies of free-ranging hibernating bears, the only large hibernators, are scarce. Yet owing to their low surface-to-volume ratios, bears experience different energetic challenges, specifically slower cooling rates and an inability to rely on dropping body temperature for metabolic rate reduction, as do small mammals [9].

Collecting data from free-ranging bears represents additional challenges. First, assessing hibernation patterns in response to climate variability requires long-term monitoring of environmental and phenological covariates to compare with physiology and behavior. Second, evaluation of causative relationships between environmental cues and physiological variables are required to assess drivers of behavior. Third, methods are required to accurately determine hibernation duration.

We sought to overcome these three challenges by developing novel applications of both behavioral change point analysis, BCPA [10], to accurately estimate den entry and exit dates, and convergent cross-mapping (CCM)[11], to assess causation between given biotic and abiotic time-series variables. CCM works on the premise that the Pearson correlation (ρ) coefficient increases significantly with increasing length (L) of the period of association. We applied these methods to a unique longitudinal data set collected from 14 free-ranging Scandinavian brown bears (*Ursus arctos*) over a period of three years, to examine the interplay between ecological, behavioral, and physiological time-keeping mechanisms involved in the hibernation processes of free-ranging brown bears.

## Methods

### Study area

The study area encompassed about 21,000 km^2^ in south-central Sweden (61°N, 15°E, Additional file 1: Figure S1). The topography in this region is rolling hills, with <10 % above 750 m above sea level. The area is forested and dominated by Scots pine (*Pinus sylvestris* L.) and Norway spruce (*Picea abies* H. Karst) [12]. The area is heavily used by hunters with dogs, during both the moose (*Alces alces*) hunting season (September–October) and the bear hunting season (21 August to 15 October or until the quota is filled). This hunting period overlaps with the predenning period [13].

### Data collection

Fourteen bears (8 males, 6 females, 2–8 years old, 30–233 kg) were captured by darting from a helicopter from April to June 2010, 2011, and 2012 [14, 15]. The bears were fitted with collars, which included a global positioning system (GPS), dual-axis motion sensors to monitor activity, described in detail previously [16], very high frequency (VHF) transmitters, and a Global System for Mobile modem (Vectronic-aerospace, Berlin, Germany). GPS positions were recorded every 30 min. The offspring of marked females were followed from birth; otherwise, age was determined by counting the annuli of a cross-section of the premolar roots [17]. We excluded six females that became pregnant during the study (also excluded from above total; these have been reported elsewhere [16]).

All implants were sterilized with ethylene oxide gas (Anaprolene AN74i 60 L, Andersen Europe, Kortrijk, Belgium). We programed temperature loggers (DST Centi, Star Oddi, Gardabaer, Iceland, 46 x15 mm; 19 g) to record body temperature (T_b_) at intervals ranging from 1 to 30 min, depending on other ongoing studies. With a memory capacity of 175,000 temperatures, the data loggers could record T_b_ every 3 min for up to 1 year [18]. Each temperature logger was individually calibrated by the manufacturer for 41 set points over the range 5 °C to 45 °C with a guaranteed accuracy of ±0.1 °C for the full temperature range one year post calibration. The equipment used for the calibration of the loggers, as stated on the calibration certificate from the manufacturer, is a Hart 7012 temperature bath and the reference measurements are conducted with a Hart 1504 thermometer and a Hart 5610–9 thermistor probe with combined absolute accuracy better than ±0.010 °C. Each set point measurement was taken when the temperature was stable within 0.001 °C. We surgically implanted temperature loggers into the abdomen, as previously described [14]. In some cases, temperature loggers were surgically removed and replaced in conjunction with a change of collar (at intervals of 1–2 years).

We used insertable cardiac monitors (Reveal DX and XT; Medtronic Inc., Minneapolis, Minnesota, USA; 8 mm x 19 mm x 62 mm; 15 g). We surgically implanted them peristernally on the left side between the muscle and subcutaneous fat and closed the incision using 2–0 monofilament glycomer (Biosyn Corporation, Carlsbad, California, USA). The device reported daytime mean heart rate (HR) (08:00–20:00) and nighttime mean HR (0:00–04:00) and contained ECG and acceleration sensors, as described previously [19]. The device determined heart rate variability (HRV) by calculating 5-min medians of ventricular intervals in milliseconds during sinus rhythm and computing the standard deviation of those medians over each 24-h period (SDANN: standard deviation of all the five-minutes NN interval means; the term "NN" was used in place of RR, to emphasize that the processed beats were "normal" beats, which means that extra systolic beats were not included).

We obtained ambient temperature (T_A_) and snow depth data for all of Sweden (620 weather stations) from the Swedish Meteorological and Hydrological Institute (SE-601 76 Norrköping, Sweden). This data were interpolated to a 1-km scale, which resulted in a daily map of T_A_ and snow depth for the entire country. From these maps, we extracted the local temperature at each bear location. Photoperiod was defined as the time between sunrise and sunset and was calculated for the same latitude (61° 6’ N) using the R-package Geosphere [20].

### Den entry and exit dates

We estimated den entry and exit dates using BCPA on the GPS data. This method sweeps through changes in the magnitudes of animal movement speeds and changes in direction, to detect points of speed and directionality change [10]. Because we were interested in the dates on which bears entered and exited the dens, we calculated mean daily location, which we used to estimate the velocities and changes in direction of the study animals at the daily scale. The method we used first computed the velocity *(V)* and changes in direction *(Ψ)* from the data and then decomposed these results into orthogonal components of persistence velocity *V*_*p*_*(t)* and directional change *V*_*t*_*(t)* defined as:

$$ {V}_p\left({T}_i\right) = V\left({T}_i\right)Cos\left(\varPsi \left({T}_i\right)\right)\kern6.25em {V}_t\left({T}_i\right) = V\left({T}_i\right) Sin\left(\varPsi \left({T}_i\right)\right) $$

where *V*_*p*_ is the tendency and magnitude of a movement to persist in a given direction, and *V*_*t*_ is the tendency of movement to head in a perpendicular direction in a given time interval. Thus, we could estimate mean velocity (*μ*), variation (*σ*), and directional persistence (*ρ*). *ρ* is the first-order autocorrelation (also called the autocorrelation coefficient) at a measured time lag one. A more detailed description has been reported previously [10].

This method identifies change points by the simultaneous changes in *μ*, *σ,* and *ρ*. We considered a bear to have entered the den in the autumn on the date that the values of these parameters became 0, and we considered the bear to have left the den on the date that the values became positive in spring (Additional file 1: Figure S4). The advantage of this method is that it provides easily obtainable and analyzable parameters, which contain more information than simple estimates of speed and turning angles, and controls for differences in the distance traveled by animals. Thus, the speed and turning velocities were comparable across individuals. We applied this method to each individual bear to identify the den entry and exit dates. We then overlaid the GPS position of the bear on this specifically identified date with the GPS location of the den to confirm if the bear was at the site. Some bears entered or exited the dens several times before the final entry or exit, as detected by the BCPA.

After estimating the den entry and exit dates, we aggregated (using means) the activity, T_b_, and HR data sets at the daily scales, to compare to movement data and to infer simultaneous daily changes in these variables and their values on the days of den entry and exit. We constructed plots for each bear showing T_b_, HR, activity, displacement (change in GPS positions), and T_A_.

### Generalized additive mixed models (GAMMs)

We used GAMMs to identify the days of increases and decreases in T_b_ and other variables. We modeled these changes for all recorded variables (T_A_, T_b_, HR, photoperiod, and activity) using GAMMs in the mgcv package in R [21].

For the variables measured at the individual animal level for multiple years (T_b_, HR, HRV, and activity), we fitted the GAMMs with a spline of ‘Day of the year/Julian day’ as a fixed effect and ‘animal-year ID’ as a random effect. For variables measured at the daily level, but for multiple years (T_A_, snow depth, and photoperiod), we fitted the GAMMs with the spline of ‘Julian day’ as a fixed effect and ‘year’ as a random effect. We used GAMMs to fit trends to the variables, because all of the variables showed nonlinearity (increasing and decreasing at different times of the year). We also explored the autocorrelation function (ACF) and partial autocorrelation function (PACF) for the variables to account for temporal autocorrelation and decided to use autocorrelation moving average correlation structures (corARMA) [21], because these autoregressive (AR) models fit the data better (tested using ANOVAs). After fitting the models, we determined the periods in which the variables were significantly increasing or decreasing. We determined these periods by computing the first derivatives of the fitted trends (GAMMs above). We used a method of finite differences, where we calculated the values of the fitted trend at a grid of point over the entire data set. We then swept through the grid by one point and recalculated the values of the trend at the new locations. The differences between the two sets of values provided a slope of the trend at that particular point, and the trend was calculated at 365 points. We then overlaid the estimated dates and periods of increase and decrease over the fitted models.

### Relationship between physiology and the external environment

To identify the predictors of changes in T_b_, HR, and activity, we again used GAMMs. We aggregated all of the variables measured at the individual animal level (T_b_, HR, and activity) to a mean daily value for that variable across individuals. We then merged all the variables (T_A_, T_b_, HR, snow depth, photoperiod, and activity) into a data set at a daily scale for multiple years. We then created three sets of GAMMs using snow depth, T_A_, and photoperiod as explanatory variables to predict changes in T_b_, HR, and activity and used year as a random effect. We again used the corARMA structure to account for temporal autocorrelation for both sets of models. To test for which ecological variable drove the changes in physiological variables during entry and exit, we split the year into two halves and ran the same GAMMs, as described above.

To determine the contribution of abiotic and physiological factors to dates of den entry and exit, we separated den entry and exit periods and set the entry/exit dates as time zero. We then aligned all data on time zero and determined the dates of significant increases or decreases in each parameter by fitting GAMMs (Fig. 2). We superimposed the dates of significant changes on yearly average environmental variables (i.e. T_A_, snow depth, and photoperiod) as a proxy of the den climate. This enabled us to determine the sequence of environmental and physiological events that were associated with den entry and den exit.

### Casual relationships

To identify the causal relationships between the monitored environmental and physiological variables, we used a convergent cross-mapping approach devised to detect causal relationships between pairs of processes represented by time series [11]. We used this approach to test for causal relationships between T_A_, T_b_, HR, and SDANN. We tested specific relationships during critical periods of interest (Table 1). The periods were based on patterns observed during data exploration. Note that we used GAMMS to determine correlates of den entry and exit, whereas CCM was used to determine causation between pairs of variables.

**Table 1:** Cross Convergent Mapping (11) analyses of the causation between different ecophysiological variables. Standard deviation of all the five-minutes normal heart beat interval means (SDANN), heart rate (HR), ambient temperature (T_A_), and body temperature (T_b_). The library length of the association ‘L’ was set to 100 for all analyses and ρ is the range of Pearson correlation coefficients for the tested relationship during the defined period (Column 1)

Period	Decription	Relationship tested	*P*- value	ρ
Period 1	From 25 days before den entry (the day activity starts to drop) until den entry	SDANN causes HR	0.1	0.1–0.7
Period 2	30 days following the date of den entry	SDANN causes T_b_	0.05	0.8–0.9
Period 2	30 days following the date of den entry	T_A_ causes T_b_	0.1	0.3–0.4
Period 3	Between 63 to 25 days before the date of den exit	T_A_ causes T_b_	0.06	0.7–0.8
Period 3	Between 63 to 25 days before the date of den exit	SDANN causes T_b_	0.9	0.1–0.5
Period 3	Between 63 to 25 days before the date of den exit	T_b_ drives HR	0.07	
Period 4	From 25 days before den exit to the den exit date	SDANN causes T_b_	0.01	0.2–0.8
Period 4	From 25 days before den exit to the den exit date	SDANN causes HR	0.03	0.3–0.8
Period 4	From 25 days before den exit to the den exit date	T_A_ causes T_b_	0.3	0.1–0.4
Period 5	From 10 days before den exit to the den exit date	Activity causes T_b_	0.05	0.5–0.8

A causal relationship was detected when the Pearson correlation coefficient ρ was significantly greater than zero for a large library length (defined as L), and ρ increased significantly with increasing L. The method was comprised of three steps; selecting an embedding dimension value that should correspond to a high predictability for a time step into the future. The predictive power should drop as the length of the prediction time step increases. Bootstrapping was then used to increase the precision of p, the number of iterations were increased in order to reduce Monte Carlo stochasticity, and iterated until mean and S.D, stabilized [11].

## Results

**Fig. 1:**
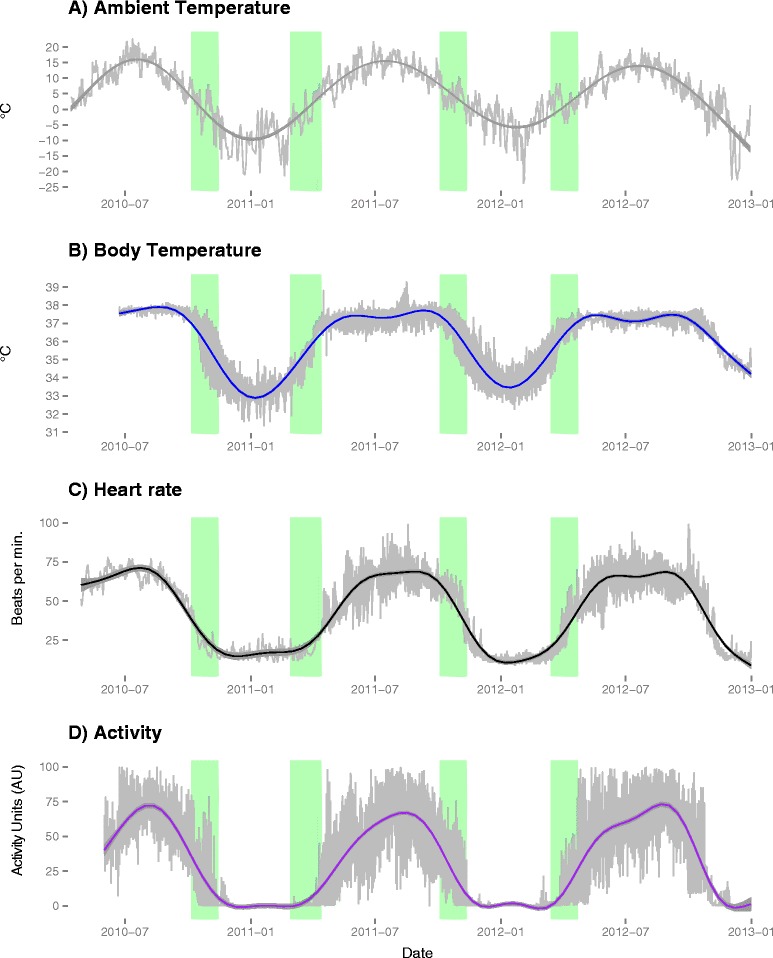
Average of the daily mean values for ambient temperature (**a**) bear body temperature (**b**), heart rate (**c**) and activity level in accelerometry units (**d**) for 14 individual free-ranging brown bears in central Sweden collected over 3 years. The X-axis indicates the time of year. Green vertical bars indicate the den entry and exit periods. The width of the green bars denotes the range of den entry and exit dates across all individuals. Trend lines were calculated using GAMMs

**Fig. 2:**
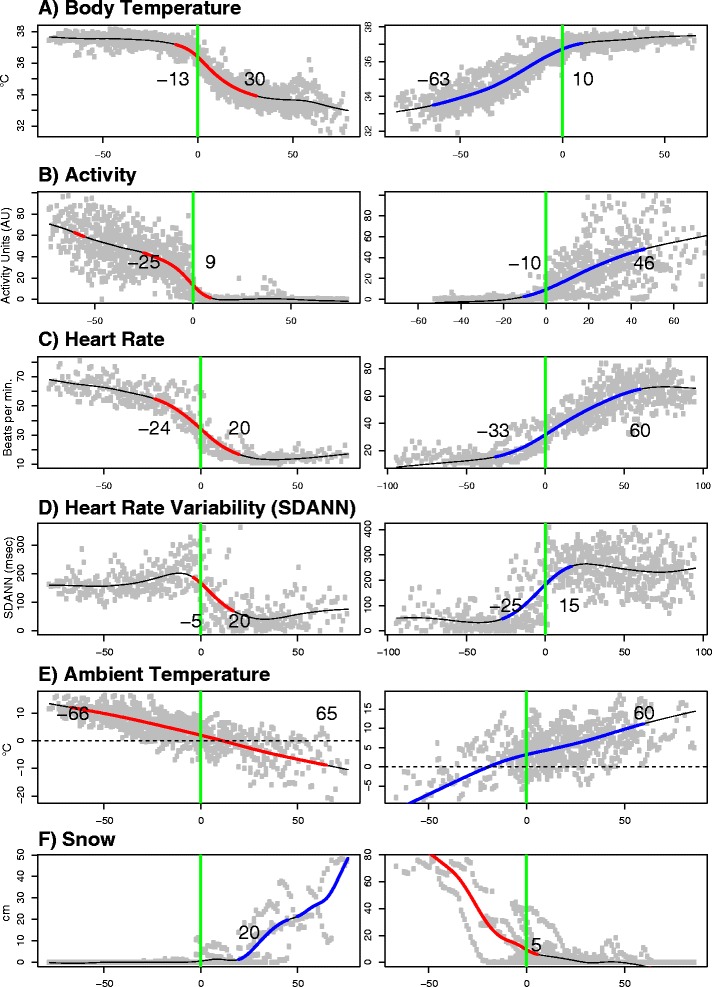
The relationship between physiological parameters of brown bears in Sweden and the dates of den entry and exit indicated by time zero. The data have been aligned to the date of den entry and exit (blue vertical line) to determine the sequence of physiological **a**: body temperature; **b**: Activity; **c**: heart rate; **d**: SDANN, and environmental **e**: Ambient temperature; **f**: Snow levels events. The red lines indicate when the variable began to decrease during den entry and increase during den exit, with the number of days from the entry/exit indicated. This is based on generalized additive mixed models (GAMMs). The gray points indicate the daily average value for each of the 14 individuals

The longitudinal design of the study resulted in a combined total of 38 years of bear data (data summary, Fig. 1, example individual Additional file 1: Figure S2, change points for individual variables Fig. 2 and Additional file 1: Figure S3). According to the BCPA (Additional file 1: Figure S4), bears entered the den during the months of October and November (median date 30 October) and exited from 21 March to 6 May (median date 6 April). Denning behavior was highly variable among individuals, but none of the bears changed dens during the study period. Both entry and exit date variability could be explained by T_A_ variation between years (Additional file 1: Table S1), with the warmer winter associated with later entry and shorter hibernation (winter 2010–11 hibernation, mean ± SD: 175.3 ± 22.4 days versus 151.2 ± 15.3 days for winter 2011–12; t-value=6.78, p=0.03).

In order to determine the ecophysiological triggers of denning, we corrected for the between-individual variation in denning behavior by aligning all data to individual den entry and exit dates. We then referenced den entry and exit dates as zero time points for all measured parameters. Mean and standard error of the physiological variables at den entry and exit are presented in Additional file 1: Table S1. We observed that T_b_ (mean ± S.E.; 37.2 ± 1.6 °C) started to drop on average 13 days prior to den entry (Fig. 2) and activity and HR decreased 25 and 24 days before den entry, respectively. We used the SDANN index as an indirect measure of sympatho-vagal balance [22]. We observed that SDANN was not related to HR in a way that could be explained by the models used in this study (SDANN drives HR, p=0.1, Table 1). SDANN declined only five days before den entry (Fig. 2). It is difficult to assess the contributions of the sympathetic and parasympathetic nervous systems to the overall autonomic balance during entry into hibernation for bears and we cannot exclude that alterations in autonomic nervous system activity may be driving processes that we would not be able to detect by assessing SDANN alone. However the drop in SDANN observed in this study likely reflected metabolic depression due either to increased parasympathetic activity or decreased sympathetic activity (or both).

Den entry occurred when T_A_ was 1.03 ± 0.95 °C (mean ± S.E.) and when snow had started to settle (Fig. 2 and Additional file 1: Figure S3, Additional file 1: Table S1). Activity stabilized nine days after den entry, but it took 20 days for HR and SDANN to reach a plateau. T_b_ stabilized at 33.8 ± 2.1 °C during the first 30 days after den entry and causation analysis by CCM showed that SDANN was a driver for changes in T_b_ (SDANN drives T_b_, p < 0.05, Table 1) to a larger degree than T_A_ (T_A_ drives T_b_, p < 0.1, Table 1).

### Environmental drivers of physiology

On an annual scale, the only recorded environmental parameter that was significantly correlated with T_b_ was the average daily T_A_ (F=14.76, p < 0.001, Additional file 1: Table S2). Neither average daily snow depth nor photoperiod significantly influenced T_b_ or HR (Additional file 1: Table S2). The average daily activity level was positively affected by snow depth and T_A_ (interaction of snow depth and T_A_, F=2.63, p < 0.001) (Additional file 1: Table S3).

During the den entry period, T_A_ and its interaction with snow depth were significantly correlated with T_b_. In contrast, during the exit period, T_A_, snow depth, and their interactions were not associated with T_b_ (Additional file 1: Table S3). None of the environmental variables were significantly correlated with HR for either den entry or exit (Additional file 1: Table S2 and S3). The difference between T_b_ and T_A_ gradually decreased during the first period (Additional file 1: Figure S5).

**Fig. 3:**
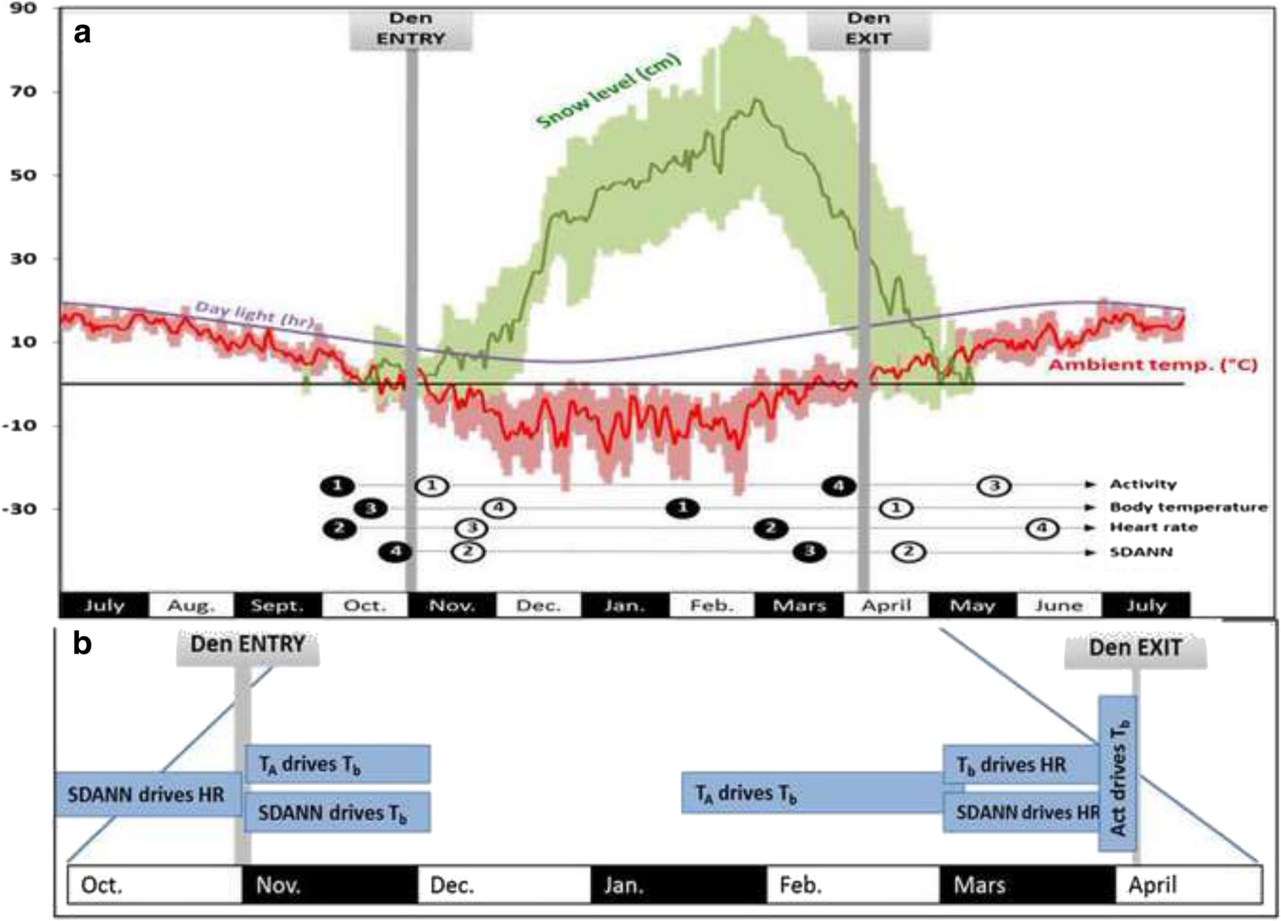
Chronology of physiological events (average change points calculated by GAMMs and shown in Fig. 2) superimposed on abiotic parameters during the annual cycle of brown bears in Sweden. The numbers below the X axis represent the sequence of events preceding den entry (black circles) and the sequence of variables reaching winter levels (open circles). For den exit, the numbers indicate the sequence of the variables beginning their transition to summer levels (black circles) and the end of the transition to the active period (open circles). Den entry occurred when the ambient temperature average was close to 1 °C, shortly after the first snowfall. Den entry occurred when ambient temperature was around °C, although body temperature (T_b_) began increasing long before the heart rate (HR), SDANN and physical activity began

T_b_ was the first physiological parameter to change during arousal from hibernation. T_b_ started to rise gradually as early as 2 months prior to den exit (mean ± S.E. from 33.2 ± 0.8 °C at 63 days before exit). HR started rising a month later and was followed by SDANN (20 days before exit) and activity (10 days before exit). We observed two patterns of thermoregulation during arousal from hibernation. From the rise of T_b_ to the rise of the SDANN (the period beginning 63 days and ending 25 days before den exit), causation analyses revealed that T_A_ influenced T_b_ (p=0.06, Table 1, Fig. 3) and T_b_ influenced HR (p=0.07). During that period, SDANN did not influence T_b_ (p=0.98). The second period of hibernation arousal was observed when SDANN started to rise (25 days prior to exit). At this time SDANN caused a rise in both T_b_ (p < 0.01) and HR (p=0.03). During this period, T_b_ was no longer associated with T_A_ (p=0.3, Fig. 3). Activity increased 10 days prior to den exit; its contribution to the return to euthermia was confirmed by the CCM, finding that it caused a rise in T_b_ (p=0.05). Den exit occurred when T_b_ had almost reached euthermia (mean ± S.E. 36.7 ± 0.15 °C) and T_A_ was 3.7 ± 1.3 °C. It took 10 and 15 days, respectively, for the bears to stabilize their T_b_ and SDANN after den exit. It took another month before HR and activity had stabilized. Fig. 3 combines these results and summarizes the different environmental and physiological drivers of brown bear denning in this population.

## Discussion

During the den entry period, T_b_ and activity level appeared to be influenced by environmental factors, such as T_A_ and snow depth. T_A_ declined before activity levels and both parameters preceded the decline in T_b_ and the first snow event (HR and the SDANN both declined later than these variables). The den emergence process began with increases in T_A_, T_b_, HR, SDANN, and activity level, in that order, apparently independently of snow depth. The observation that SDANN increased only 13 days after the increase in HR (i.e. a gradual shift in autonomous nervous system balance) suggested that the first increase in HR was adaptive thermoregulation (through a temperature-dependent increase in metabolic activity). Although this is well described in small hibernators and even in humans during induced therapeutic hypothermia, this observation would require further investigations.

The BCPA greatly increased our ability to estimate den entry and exit dates (Additional file 1: Figure S4). A simple evaluation of GPS positions would not have sufficed, because the transition period between hibernating and active states was drawn out, and some bears regularly or periodically returned to the denning area after emergence. We observed significant differences in the timing of den entry and exit between years, with the latest den entry dates during the warmer years. As climate change projections predict warming wintertime temperatures for the [23], shorter hibernation periods can be expected. For example, the decline in T_b_ before den entry was significantly correlated with T_A_, whereas HR was independent of the predictor variables we tested, except for day of the year. This suggests that environmental variability can affect behavioral and physiological aspects of hibernation independently in the bear.

Our results regarding timing of den entry and exit were consistent with a previous study on this population [24]; den entry and den exit dates occurred within the same ranges and males and older animals entered the dens later and exited earlier than females and younger animals [25]. Although a study on brown bears in Alaska documented a correlation between den emergence and the timing of snow melt [26], the bears in our study area had emerged from their dens either before or during snow melt (Fig. 3). Moreover, during emergence, there was no apparent impact of snow depth or photoperiod on T_b_ or HR; however, activity level was affected by both snow depth and photoperiod. This result indicated that, although snow depth was associated with a change in the behavior of the bears (e.g. den entry, consistent with the previous report [25]), it was not associated with any physiological change measured in this study. However, we found a difference in den entry from year to year, with bears entering the dens earlier in a colder year.

### Drivers of den entry

Although T_b_ was correlated with several factors during den entry (Additional file 1: Table S2), convergent cross mapping revealed the greatest causation due to SDANN, followed by T_A_ (Table 1). SDANN and HR were closely related at this stage of hibernation, likely because activity was low, eliminating activity’s confounding effect on HR. SDANN declined steeply just before den entry. Although the SDANN is not the best proxy to assess autonomic nervous system balance, we were unfortunately not able to use another index due to the duration of the experiment and the storage capacity required for a full ECG to be stored. Therefore, it is difficult to determine if changes in SDANN should be attributed to changes in the parasympathetic or sympathetic nervous system or both. Previous studies [27] have shown that hibernating bears have an enhanced respiratory sinus arrhythmia, indicating increased parasympathetic nervous system activity. Based on the literature on small hibernating mammals (e.g. [28, 29], the observed decrease in SDANN likely indicated that a predominant parasympathetic tone drives metabolic suppression and decreases in most physiological functions. In small hibernators, cooling is achieved by both metabolic depression and passive body cooling. Our data are in the line with the previously reported observation that large hibernators initially rely on metabolic depression to achieve depressed metabolic rate during hibernation [9, 30] to a larger extent than passive body cooling, although both mechanisms occur. The early rise in T_b_ in the bear is in contrast to most hibernators, with likely two involved mechanisms that cannot be dissociated but likely happen sequentially: Q_10_ effect due to slow warming with concomitant ANS activity and final warming from a massive SNA burst.

Both SDANN and HR stabilized 20 days after den entry, but it took an additional 10 days for T_b_ to stabilize, probably because of the bear’s large body mass and decreased heat exchange in the den. This is similar in sequence to that found in woodchucks (*Marmota monax*), where the decrease in metabolic rate occurred in 6 h, whereas the drop in T_b_ continued for 12 h [31], and in golden hamsters (*Mesocricetus auratus*), with metabolic rate decreasing for 3 h and T_b_ dropping for 8 h [32, 33]. However, the mechanisms of metabolic rate reduction are thought to differ between large and small hibernators [9]. Large hibernators, such as bears, are expected to rely to a greater extent on active metabolic suppression to reduce their metabolism, due to their larger body size, compared to small hibernating species, which benefit more from the Q_10_ effect in torpor. Even small marsupials (<20 g) actively suppress their metabolism during torpor [9]. Using active metabolic suppression, bears are able to reach metabolic rates (despite having a T_b_ above 30 °C) as low as small hibernators in deep (<5 °C T_b_) torpor [34].

Including SDANN in our study proved to be particularly valuable. In contrast to previous approaches [33], HR was not used to infer metabolic rate, because, this parameter is confounded by activity and stress [33] which are expected prior to hibernation with the combination of both the hunting season and den entry behavior [13]. However, activity affects SDANN to a much lesser extent [22]. By including SDANN, we avoided the confounding effect of activity on HR and had indirect access to information on the sympathovagal regulation of metabolism. One study in dogs found that HRV did not differ between slow movements, lying, sitting, or standing, but did change when a favorite toy was presented [22]. In stressful situations, dogs had consistently increased HR and decreased HRV [35]. Therefore, whereas HRV may often be connected to HR, it is more independent of movement-induced changes. Variability in HR can be caused by changes in thermoregulation, circadian rhythms, respiration, blood pressure, and both physiological and psychological stressors and can be used to evaluate the state of balance between the sympathetic and parasympathetic nervous systems [36], but does not give an overall level of either system’s activity.

T_b_ in captive brown bears was reported to decline gradually over 5 weeks from the date that food and water were removed [37]. Our finding that changes in T_b_ began long before changes in HR suggested that previous studies focusing on captive bears with an artificially defined end of the food/water season might not represent the actual sequence of events in the wild. In our study, SDANN declined steeply just before den entry. This change was probably associated with the enhanced respiratory sinus arrhythmia previously reported to occur in hibernating bears [27]. Based on the literature on small hibernating mammals, the observed decrease in SDANN suggested that a massive parasympathetic tone, likely with a reduction in sympathetic activity, drives metabolic suppression and decreases in most physiological functions. The role of SNS in thermogenesis and cardiovascular control has been the topic of a number of experiments starting more than 60 years ago [38]. In studies of small hibernators, the initial fall in HR during entry into hibernation is due to parasympathetic activation and the exit due to SNS activation [39]. Treatment with atropine, an inhibitor of parasympathetic pathways, prevented 13-lined ground squirrels (*Citellus tridecemlineatus*) from entering hibernation [40]. Our results are in line with those from previous studies and suggest that increased parasympathetic activation plays a key role in the reduction in HR at den entry in bears as well, but does not rule out potential decreases in sympathetic nervous system activity.

### Drivers of den exit

Although den exit was not correlated with either T_A_ or photoperiod, the bears exited the dens at T_A_ of 3.7° ± 1.3 °C. A bear den is not an adiabatic shell, however, the inside air temperature could easily rise, depending on the type of den (ant hill, under rocks or nests [41]). The fairly narrow range of T_b_ between bears on the day of exit (36.7 ± 0.15 °C, Additional file 1: Table S1) suggested that the bears exited when they reached a specific set point. At T_A_ of > 0 °C, water also could start draining into the den, causing the bear to become uncomfortable and leave the den. American black bears (*Ursus americanus*) in artificial dens have a mean lower critical T_A_ of 5 °C, below which the bears’ thermal conductance increased [42]. This suggests that the bears’ cue to exit the den was that they became too warm when the temperatures rose in springtime or that they were seeking more optimal temperatures outside the dens.

That T_A_ does not drive T_b_ during the phase before exit (period 5, Table 1) might be due to the adaptive thermoregulation that occurred over several months, making the T_A_ immediately around the day of exit less important. It could also be that the den temperature was more relevant, as the bears exited when T_A_ reached approximately 3.7 ± 1.3 °C (Additional file 1: Figure S1), nearing the lower critical temperature for established for black bears and polar bear (*Ursus maritimus*) cubs [42, 43], possibly because the den temperature was above thermoneutrality.

T_b_ started rising 2 months prior to exit, whereas HR rose a month later, and was followed by SDANN (20 days prior to exit) and activity (10 days prior to exit). During the first part of arousal, the causation analysis revealed that T_A_ caused T_b_ and T_b_ caused HR. There was no causal relationship between SDANN and T_b_, but a different trend emerged 20 days before exit.

The gradually decreasing difference between T_b_ and T_A_ during the first period (Additional file 1: Figure S5), suggests that bears were thermoregulating at a lower thermoregulatory set point during hibernation. This is consistent with recent findings from captive bears showing a negative relationship between den temperatures and hibernating metabolic rates [42]. Then, SDANN started rising and may have caused T_b_ and HR to rise, likely via an increase in sympathetic nervous system activity, a decrease in parasympathetic nervous system activity, or a combination. At this stage T_b_ lost its causal association with T_A_ although T_b_-T_A_ remained stable during the second period, suggesting that euthermic metabolism was reestablished later by active thermogenesis, likely involving the sympathetic nervous system. The exact roles of the sympathetic and parasympathetic nervous systems in this process can only be assessed by direct measurements of sympathetic and parasympathetic nervous system activity in free-ranging conditions, which would be difficult to conduct.

This second phase of den exit was driven by SDANN, with SDANN driving T_b_ (p <0. 01). When SDANN began to rise, the thermoregulatory pattern shifted. This could indicate transitioning out of hibernation i.e., sympathetic nervous system activation combined with potentially a more profound change in metabolic state [42]. Activity increased from 10 days before exit, showing a causative relationship with the increase in T_b_ (activity drives T_b_, p=0.05). Den exit occurred when T_b_ was almost at euthermia (mean 36.7 °C), nearing the lower critical temperature for bears [42]. T_b_ and SDANN stabilized quickly (within two weeks after den exit), but HR and activity took longer, indicating that the bears took longer to return to their original activity levels.

Although shivering may play a role in active thermogenesis, it occurs at the end of arousal in the species studied to date, excluding tropical hibernators [44]. Increased T_b_ allows restoration of enzyme functioning through a Q_10_ effect and contributes to restoration of muscle function. Early on, the processes start with SNS activation of the vascular system to increase body temperature and heart rate [44]. The role of SNS in thermogenesis in addition to vascular control has also been the topic of numerous investigations starting from the early studies of Lyman. Studies on American black bear in the laboratory show a role for shivering at the end of arousal [34], although our results show that it was less important in free-living conditions.

A recent study on captive American black bears found that metabolic rate was related to den temperature and showed that larger bears showed more variation in length of T_b_ cycles [42]. During experimental manipulations of den temperature, they found no direct relationship between den temperature and T_b_, although the time between peaks in T_b_ became longer at higher den temperatures. The authors suggested, based on a single bear that increased its T_b_ to 35.9 °C when the den warmed to 10 °C, that the bear may have inhibited heat dissipation mechanisms. It is not clear whether this was merely an effect of being inside an isolated den or was a physiological phenomenon. In addition, they found that the lower critical temperature varied from 1° to 10 °C, from which they concluded that the smaller bears partially compensated for higher thermal conductance with increased metabolic rate. Interestingly, they found no relationship between T_A_ and den temperatures. Although it was not possible to measure the den temperatures in our study, we would expect a correlation with T_A_, because the bears in our study were not in adiabatic shells; they were under rocks or tree roots or in anthills, with oxygen exchange varying from a small ventilation hole to large openings under rocks. We found, however, that T_A_ drives T_b_ during the first phase, and the differential between T_b_ and T_A_ decreased until the point in the spring when HRV rose. Although [42] found a negative relationship between T_A_ and metabolic rate, we conclude that this is more likely an adaptive thermoregulation allowing maintenance and slowly rising T_b_ at a minimal cost, simultaneously with the increasing T_A_.

In a previous study, the HR in captive black bears was reported to decline gradually over five weeks from the date that food and water were removed [45]. Our finding that changes in T_b_ began long before changes in HR suggests that studies on captive bears with an artificially defined end of the food/water season might not represent the actual sequence of events in the wild. Our results would have been enhanced considerably had we succeeded at measuring den temperature. Bears in this population are very susceptible to disturbance in winter [46], repeatedly changing dens after captures or capture attempts, so putting temperature loggers inside the den was not realistic.

Our novel results and the methods adapted for this analysis could impact our general understanding of how climate change influences other ecophysiological and behavioral adaptations. In this study, we demonstrate mechanisms for the entry and exit into hibernation by the brown bear in Sweden that have implications for both bear population monitoring and management. These results highlight some of the differences between the bear and small hibernators, reinforcing the importance of not generalizing results from small hibernators to bears.

This work is an example of how different types of datasets can be combined to provide coherent ecophysiological timeseries with potential applications for other ecophysiological and adaptation studies beyond hibernation. Other time-series datasets that could be analyzed in a similar way include phenological and reproduction data on different organisms that are commercially important (crops) or used as indicator species for habitat/ecosystem quality measures (e.g. birds, butterflies). The results from such analyses would provide management strategies and production optimization, while minimizing ecosystem-level impacts. Besides conservation practices, our study demonstrates the importance of several physiological and behavioral characteristics that are important for studies of adaptation, in this case to winter conditions and to climate change, in the context of selection pressures for matching the start and end of hibernation with resource availability.

## Conclusions

We demonstrate that changes in brown bear T_b_ during den entry were driven by environmental factors, particularly T_A_. This indicates that a warming climate could result in later den entry. Thus, although many studies have shown that den entry and exit are related to food availability, climate change also appears to be an important factor affecting the timing of the life events of the brown bear and could pose a threat through the mismatch of important physiological cues. Consequences would be a shortening of the bears’ hibernation period and potentially prolonging the den-entry period, which has been shown to be the highest risk period for bear caused injuries to humans [13]. This should be anticipated by wildlife management agencies in areas where there is a large overlap between humans and bears.

Further, this study suggests that Scandinavian brown bears terminated their hibernation due to physiological cues. Although body temperature started to rise slowly very early in the hibernation period, it was only few weeks before exit that we observed activation of the sympathetic nervous system to restore euthermic metabolism. Body temperatures were close to euthermia when ambient temperature reached 0 °C and bears exited the dens when ambient temperature was close to the lower critical temperature. Hibernation in brown bears seems to be initiated based on environmental cues and terminated due to physiological cues.

## Ethics

All captures were approved by the Ethical Committee on Animal Experiments, Uppsala, Sweden (application #C47/9 and C7/12) and the Swedish Environmental Protection Agency.

## Additional file

Additional file 1:
**Supplemental Figures and Tables.** (DOCX 10538 kb)

## Abbreviations

HR: heart rate
HRV: heart rate variability
SDANN: standard deviation of all the five-minute NN interval means
T_A_: ambient temperature
T_b_: body temperature
