# Supplementary material for: Drivers of hibernation in the brown bear
Source: Front Zool. 2016 Feb 11;13:7. doi: 10.1186/s12983-016-0140-6 (PMC4750243; doi:10.1186/s12983-016-0140-6)
Supplement: Additional file 1: — Supplemental Figures and Tables. (DOCX 10538 kb) [file 12983_2016_140_MOESM1_ESM.docx]

**Supplemental Figures**


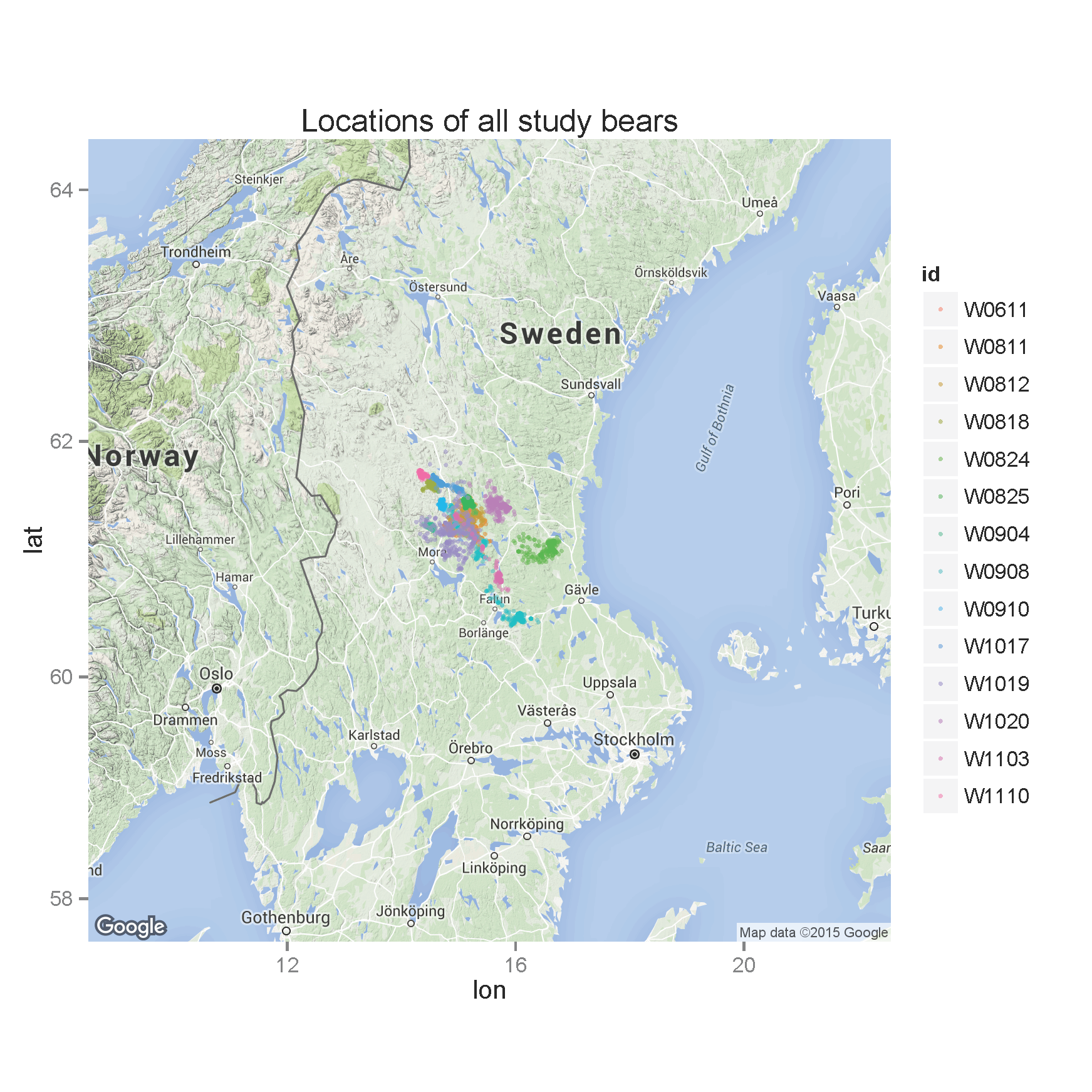


**Figure S1**. Map of the GPS positions from the studied bears in central Sweden, illustrating that they all occurred at similar latitudes.


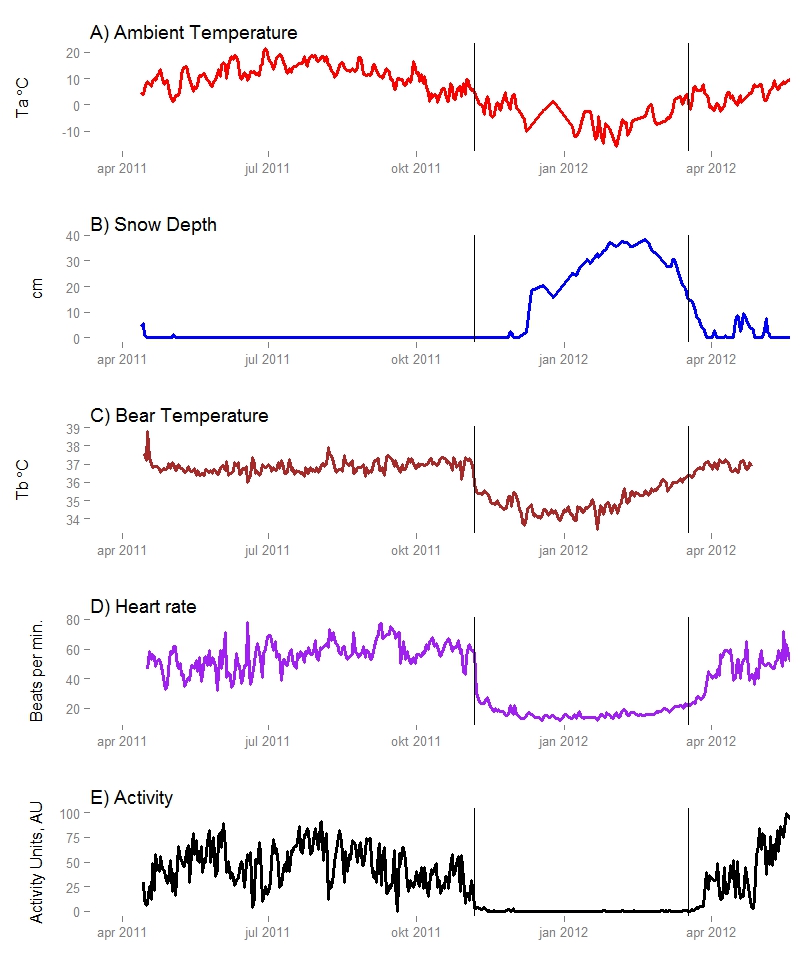


**Figure S2.** Dataset for an individual brown bear in central Sweden, demonstrating the simultaneous changes in daily mean activity, movement, heart rate, and body temperature. Vertical bars indicate the den entry and exit dates, respectively. W1019 Den Entry Date- 2011-11-13; W1019 Den Exit Date- 2012-03-26.


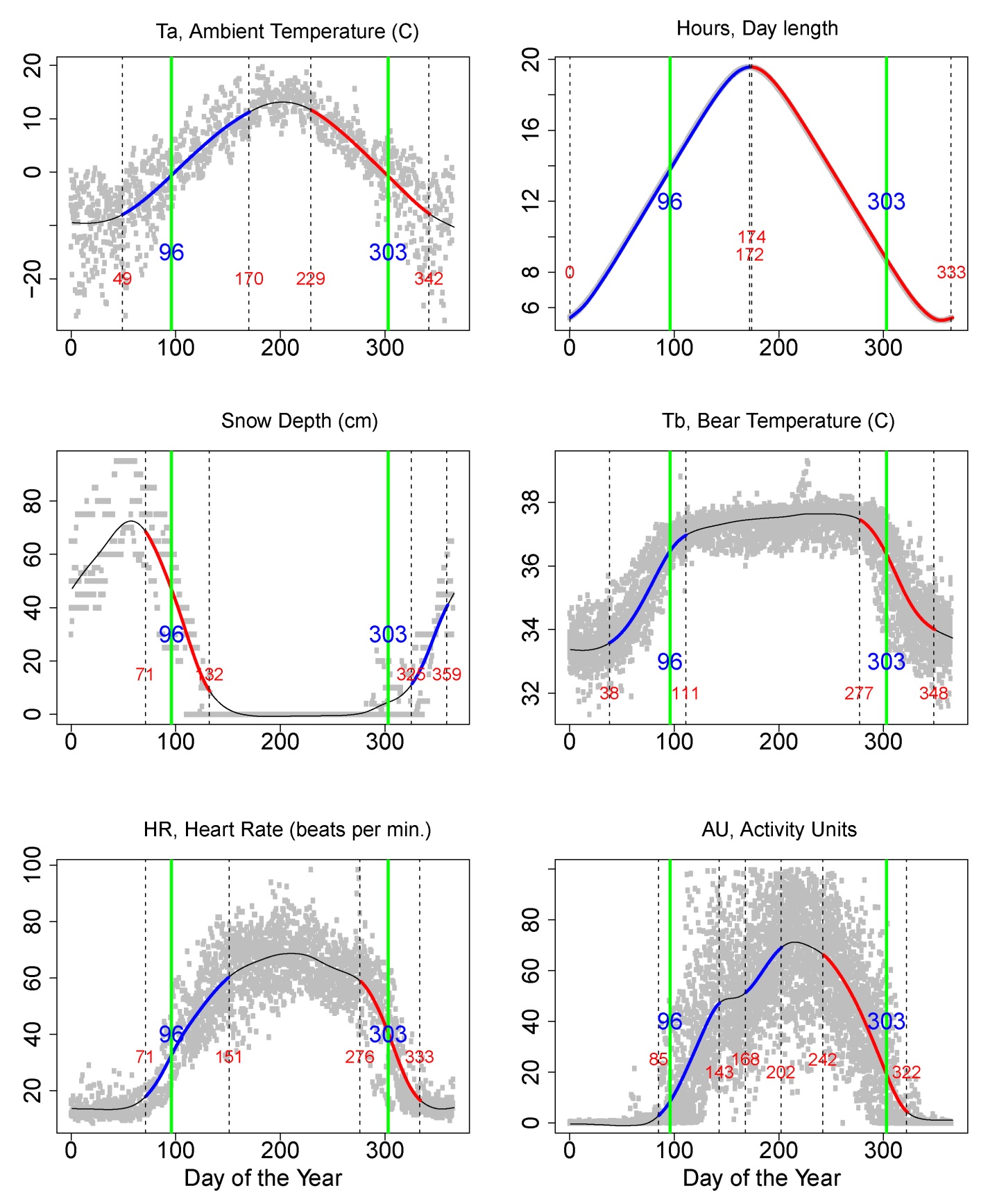


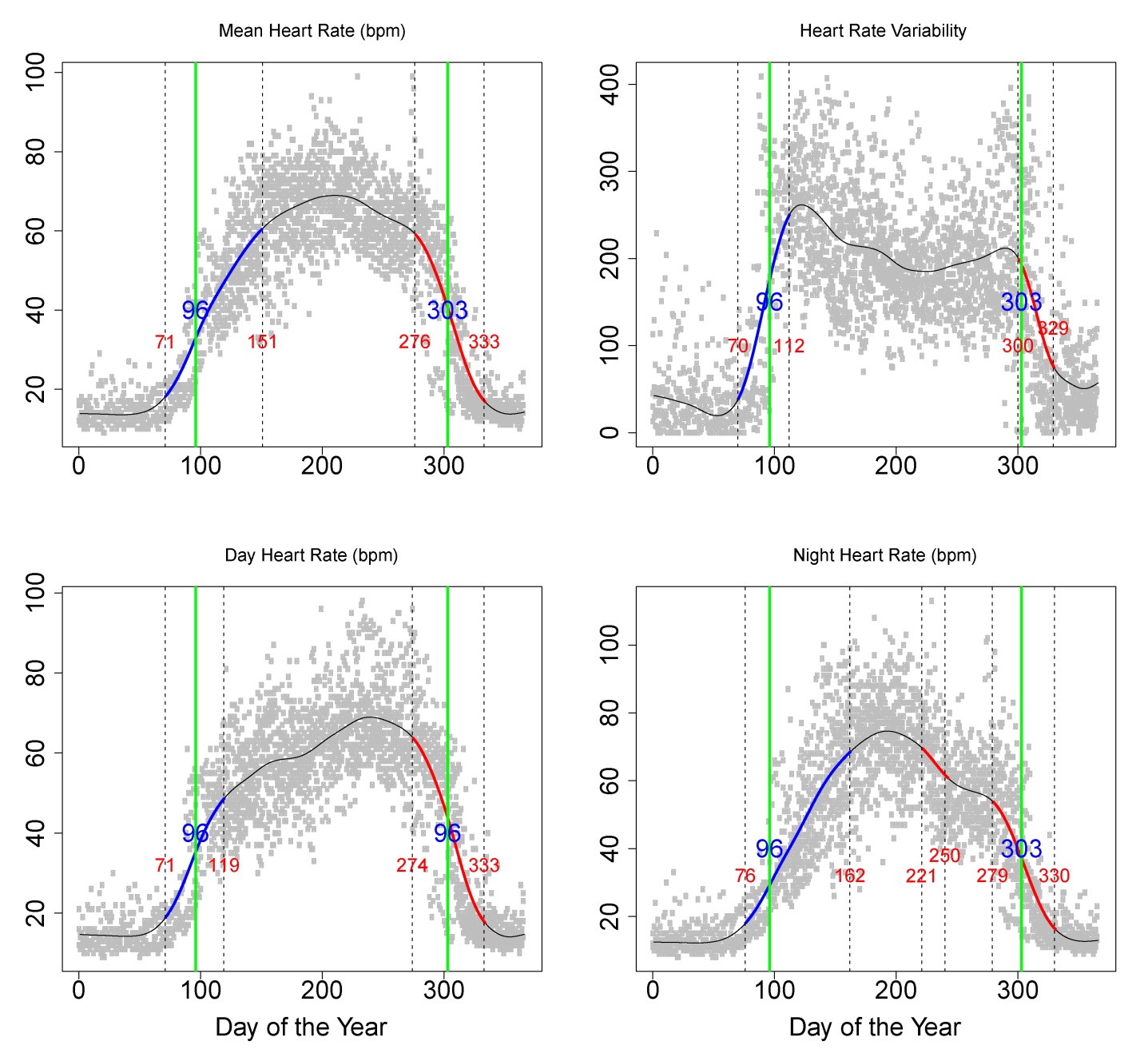


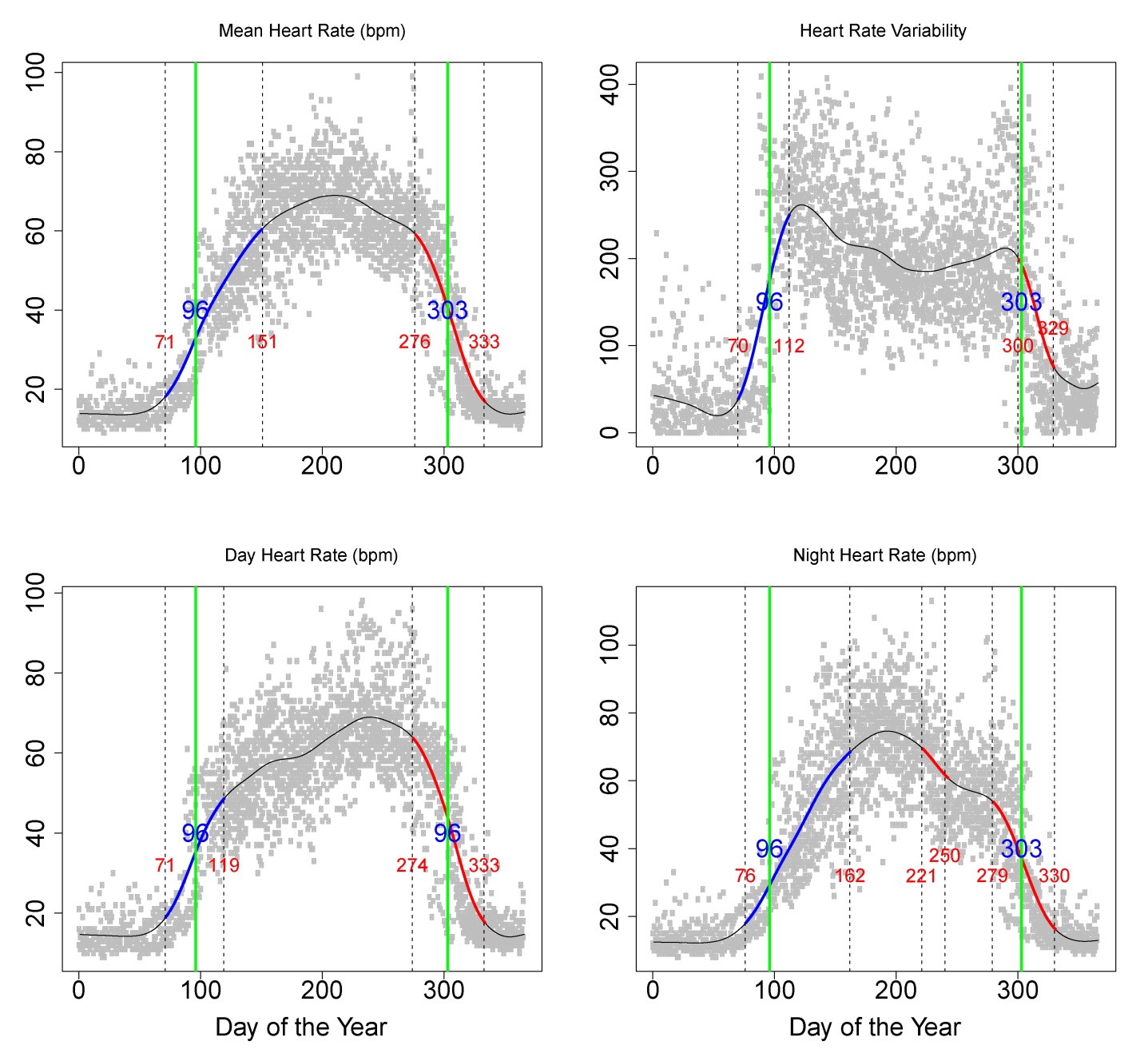


**Figure S3.** Smoothed annual variation in ambient temperature (°C), photoperiod, and snow depth (cm) on the study area in central Sweden and body temperature (°C), heart rate (bpm), and activity (AU) of the brown bears; all values were estimated using generalized additive mixed models. Green vertical bars show the median date of den entry and exit. Annotated numbers denote the Julian day. Blue numbers represent the dates of den entry and exit, and red numbers denote the date when an increase began and ended (change points). Dotted vertical bars demarcate the periods of increase and decrease in the variables, with blue overlaid curves showing the increase phase and red showing the decrease phase based on the fitted model. Gray dots show daily means for the individuals.

Entry


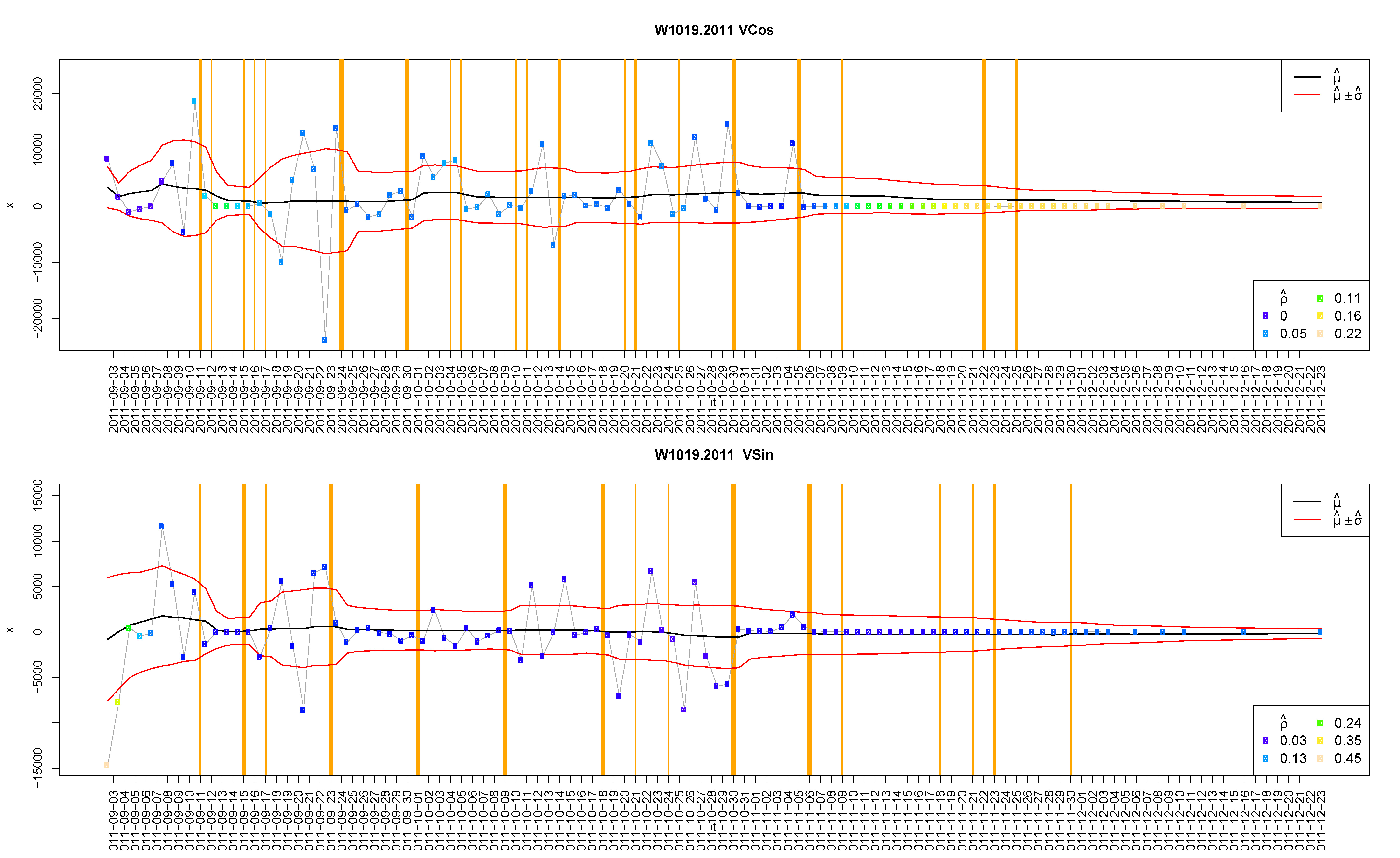


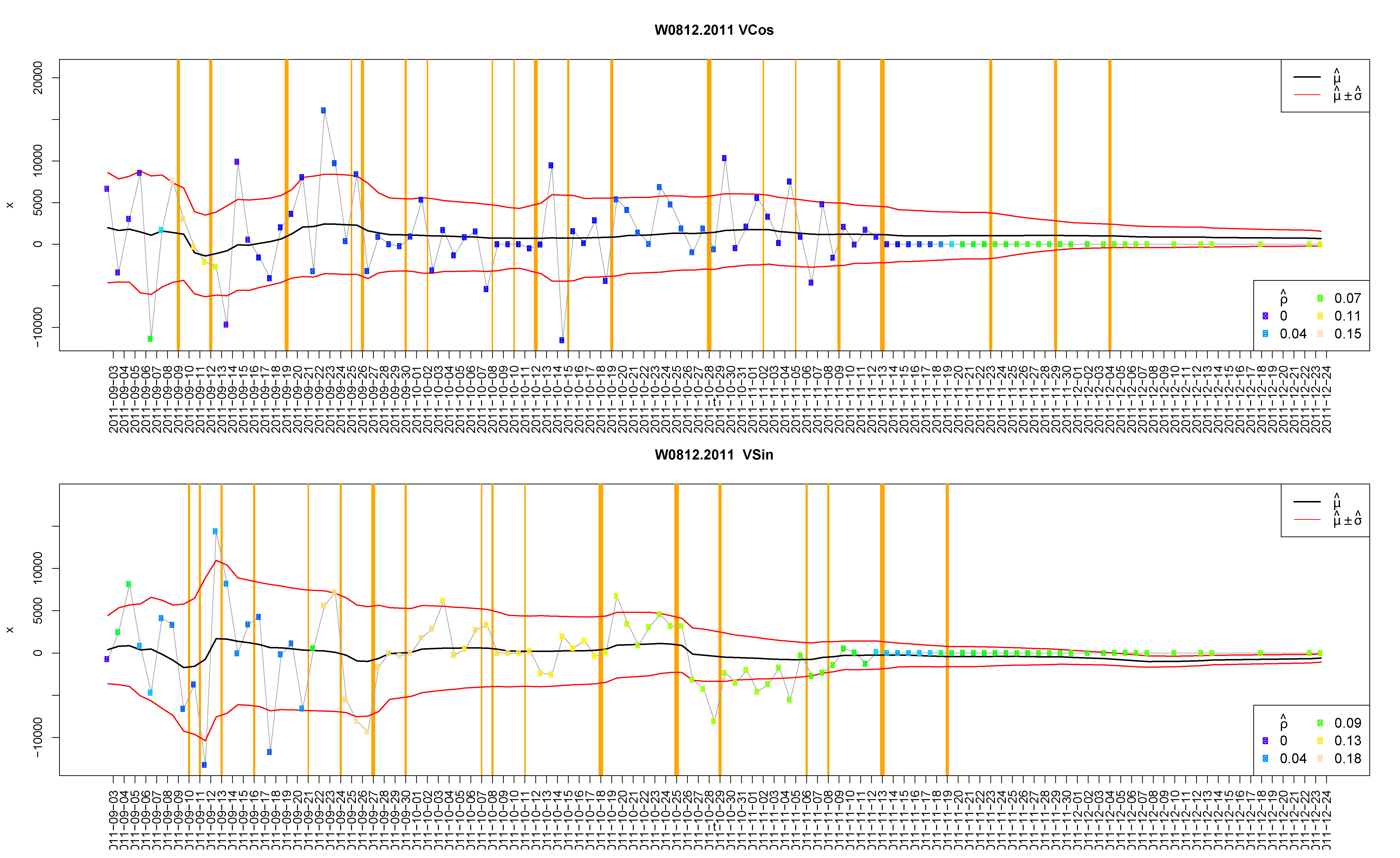


Exit


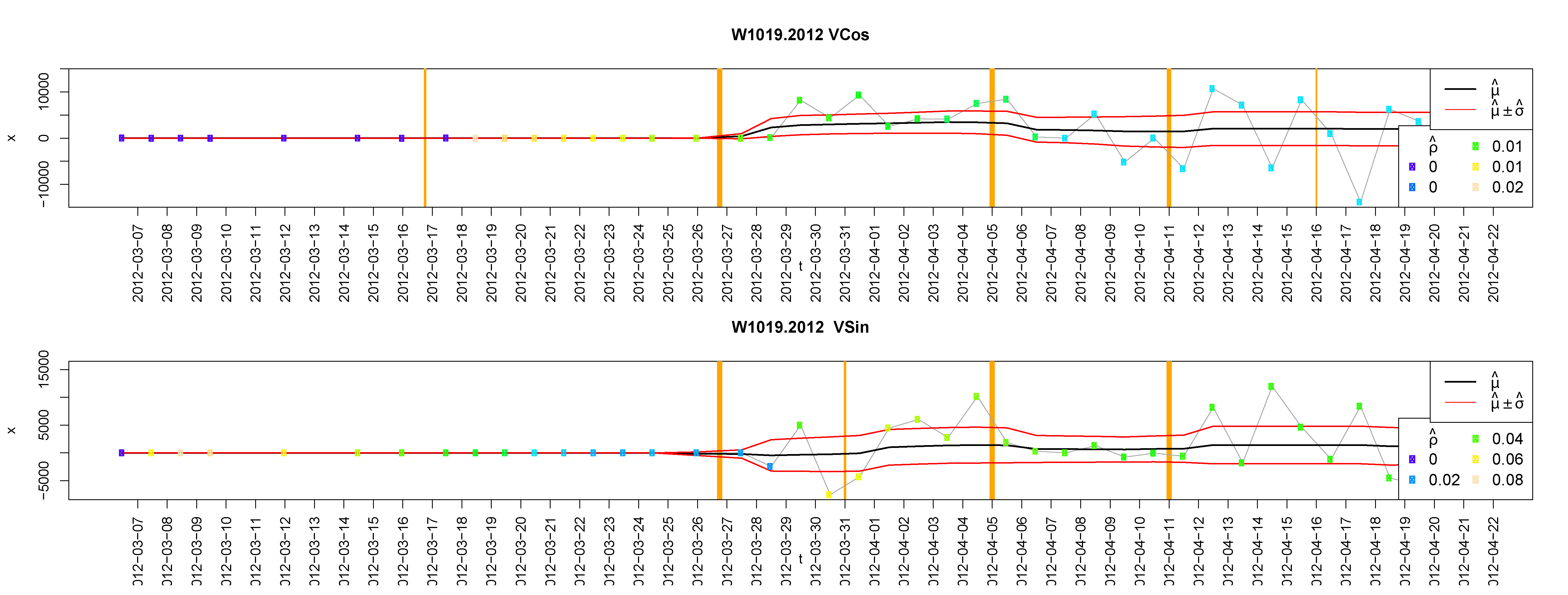


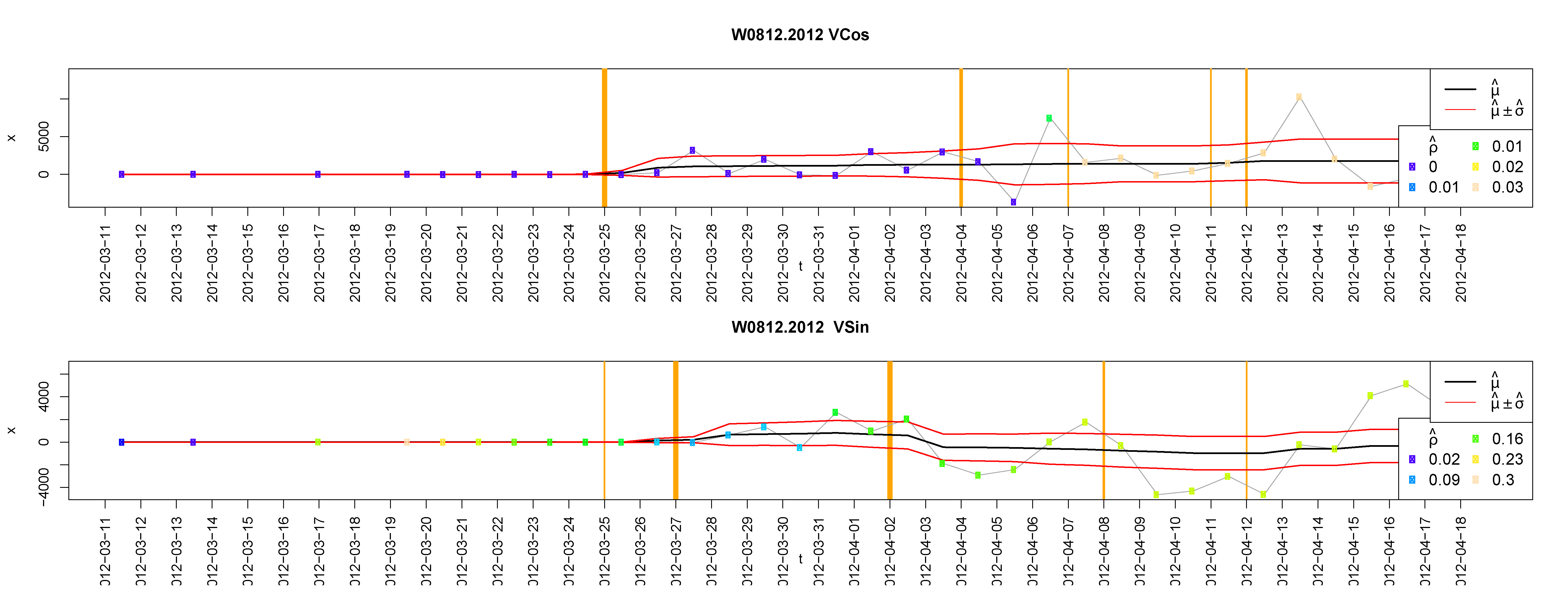


**Figure S4.** Estimation of the den entry date for two brown bears in Sweden using the GPS data for changes in velocity. The day the change in velocity became zero was considered to be the den entry date and the day the change was no longer zero, the den exit. Each dot is a bear position; the color of the dot represents the autocorrelation value (**ρ**). X axis shows the date, **μ** is the orthogonal velocity, and **σ** is the variance (Gurarie et al. 2009). Vertical bars represent the periods with significant change in behavior. The location of the last and first yellow vertical bar was selected as the entry and exit date.

**Figure S5.** Difference between mean body temperature °C and ambient temperature of brown bears in central Sweden during the days leading up to den exit, using the dataset aligned by den exit date (day 0).

**Tables**

| Den Entry |  |  | Den Exit |  |
| --- | --- | --- | --- | --- |
| T_b_ | 36.47 | 0.14 | 36.7 | 0.15 |
| T_A_ | 1.03 | 0.95 | 3.73 | 1.31 |
| HR | 37.25 | 8.98 | 28.5 | 3.92 |
| HRV | 153 | 57.3 | 185 | 53.4 |
| Act | 9.96 | 2.24 | 6 | 1.79 |
| Snow | 0.89 | 0.68 | 10.25 | 3.56 |

**Table S1:** Mean and standard error values of the eco-physiological variables during the den entry and exit of brown bears in central Sweden.

**Table S2.** Results of the annual generalized additive mixed models for body temperature, heart rate, and activity for brown bears in central Sweden. Values in blue show statistically significant variables with p values <0.01. Body temperature, heart rate and activity were included as response variables, and snow depth, day length, ambient temperature, and the interaction between ambient temperature and snow depth were included as predictor variables.

**Table S3.** Results of the half-year generalized additive mixed models for body temperature, heart rate, and activity of brown bears in central Sweden. Parameter values are presented along with their standard errors. Values in blue show statistically significant variables with p values <0.01.

**Table S4.** Correlation matrix of the monitored variables to identify the drivers of den entry and exit of brown bears in central Sweden.

|  | Bear Temperature | Activity | Mean Heart Rate | Day length | Ambient Temperature | Snow Depth |
| --- | --- | --- | --- | --- | --- | --- |
| Bear Temperature | 1.00 | 0.83 | 0.88 | 0.79 | 0.81 | -0.75 |
| Activity | **0.83** | 1.00 | 0.93 | 0.77 | 0.81 | -0.69 |
| Mean Heart Rate | **0.88** | **0.93** | 1.00 | 0.79 | 0.81 | -0.72 |
| Day length | **0.79** | 0.77 | 0.79 | 1.00 | 0.79 | -0.45 |
| Ambient Temperature | **0.81** | 0.81 | 0.81 | 0.79 | 1.00 | -0.63 |
| Snow Depth | **-0.75** | -0.69 | -0.72 | -0.45 | -0.63 | 1.00 |
